# Validation of a Low-Cost Parallel Plate Rotational Viscometer: Effect of Plate Spacing on Viscosity Measurement of Automotive Oils

**DOI:** 10.1021/acsomega.6c06689

**Published:** 2026-09-11

**Authors:** Damiano da Silva Militão, Leandro de Amorim Ratamero, Alef de Oliveira Warol, Joel Sanchez Dominguez, Joaquim Teixeira de Assis

**Affiliations:** † Departamento de Engenharia Mecânica e Energia, 631958 Universidade do Estado do Rio de Janeiro, Instituto Politécnico do Rio de Janeiro, Rua Bonfim, 25 − Vila Amélia, Nova Friburgo, 28625-570, Rio de Janeiro, Brazil; ‡ Departamento de Sistemas Integrados, 28132 Universidade Estadual de Campinas, R. Mendeleyev, 200 − Cidade Universitária, Campinas, 13083-872, São Paulo, Brazil

## Abstract

Viscosity is the most relevant property of automotive lubricants, directly influencing friction, wear, and energy efficiency of internal combustion engines. This work presents the design, construction, and validation of a low-cost parallel plate rotational viscometer, with emphasis on the systematic analysis of the effect of plate spacing (*h*) on the measured viscosity. Two commercial lubricants (SAE 15W40 and SAE 20W50) were evaluated for five plate spacings (1.25 mm to 6.25 mm) at room temperature, yielding viscosities of 0.34 ± 0.02 Pa·s and 0.39 ± 0.01 Pa·s at *h* = 6.25 mm, respectively. The experimental results were compared with literature data from Anton Paar. It is demonstrated that for *h* ≥ 5 mm, the viscosity values obtained (0.34 ± 0.02 Pa·s for 15W40) are consistent with the reference, while for *h* ≤ 3.75 mm significant deviations are observed (up to 0.78 ± 0.03 Pa·s for *h* = 1.25 mm), associated with the increase in shear rate (from 119 s^–1^ to 594 s^–1^) and possible nonparallelism effects. It is concluded that the equipment is validated for *h* ≥ 5 mm, establishing its reliable operating range.

## Graphical abstract

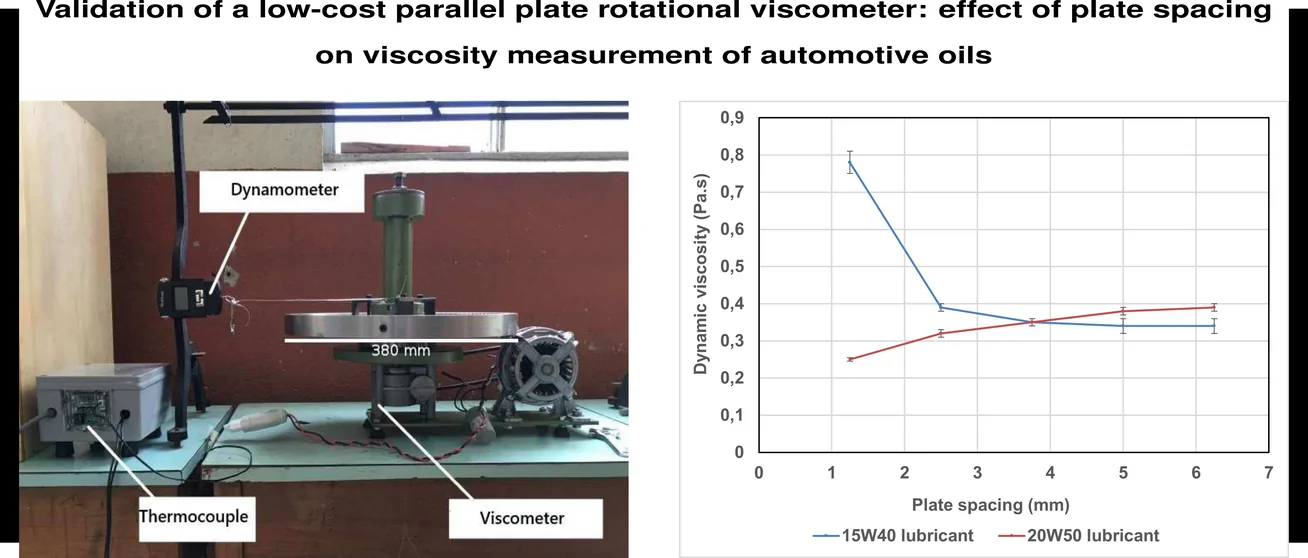

## Introduction

Viscosity is, unquestionably, the most important physical property of automotive lubricants.^1,2^ In internal combustion engines, it governs the formation of the lubricant film between components in relative motion, directly influencing friction, wear and energy efficiency of the system. Recent studies by our research group have advanced the understanding of friction mechanisms in cam-tappet systems,^4,5^ as well as the application of empirical modeling for vehicle energy efficiency estimation.^6^ It is estimated that about 15% of the energy available in the fuel is dissipated as mechanical losses in a vehicle, with friction being the main responsible.^7^

Although high-precision rotational viscometers are widely available on the market, their high costs often limit their use in educational contexts and in laboratories with budget constraints.^8^ Additionally, the literature lacks studies that present not only the design of low-cost equipment but also its rigorous metrological validation, identifying its actual reliable operating ranges.

This gap is particularly critical for educational and resource-limited laboratories, where access to such data is essential for advancing practical knowledge in fluid mechanics and tribology.

To address this gap, this work aims to contribute by presenting the following:

1. The design and construction of a low-cost parallel plate rotational viscometer;
2. The validation of the equipment through comparison with literature data from Anton Paar^3^ for the SAE 15W40 lubricant;
3. The identification of the plate spacing range that provides reliable results;
4. The application of the method for comparative characterization of SAE 15W40 and 20W50 lubricants.

A central contribution of this work is the systematic analysis of the **effect of plate spacing (** ***h*** **)** on the measured viscosity, demonstrating that for *h* ≥ 5 mm, the results are consistent with the literature, while for smaller *h*, significant deviations are observed due to the increase in shear rate and possible non-parallelism effects. This study, therefore, not only validates the equipment but also establishes its reliable operating range, an aspect often neglected in the didactic instrumentation literature (Figure 1).

**Figure 1:**
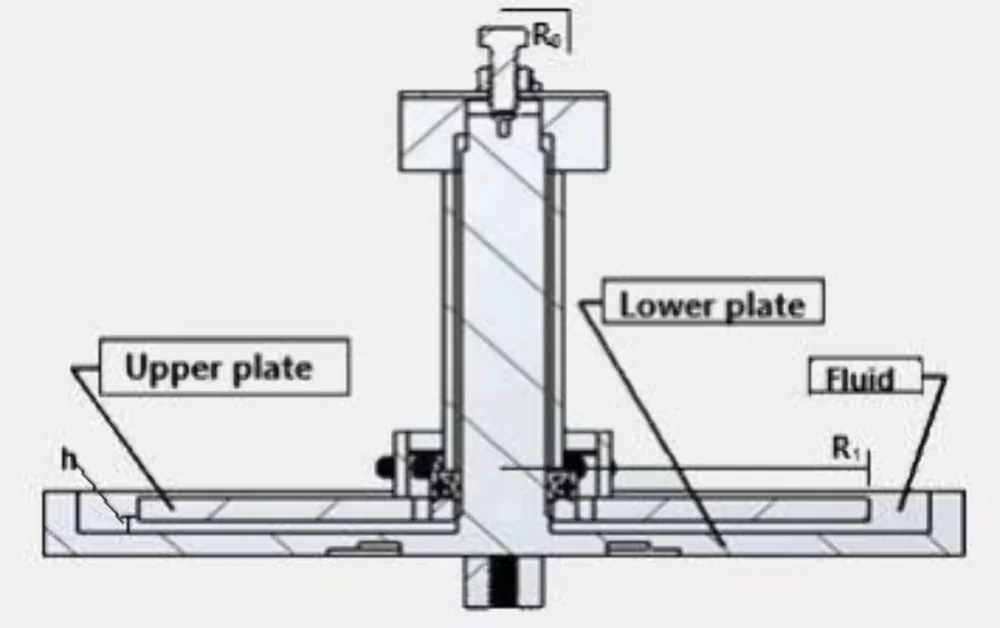
Schematic cross-section view of the constructed parallel plate viscometer, showing the flow geometry and main dimensions (*R*_0_, *R*_1_, *h*).

The originality of this work lies in the systematic identification of a reliable operating range for a low-cost viscometer—an aspect often overlooked in the development of instrumentation for education, as well as for scientific and technological research. Many commercial viscometers with a wide viscosity measurement range do not generate a significantly high shear rate in the fluid being tested. However, for internal combustion engine applications, such a pronounced effect is desirable. This device offers that capability.

The paper is organized as follows: Materials and Methods describes the experimental apparatus and methodology, including mathematical modeling and uncertainty analysis; Results presents the experimental results; Discussion discusses the findings; and Conclusions presents the conclusions.

## Materials and Methods

### Experimental Apparatus

A parallel plate rotational viscometer was constructed at the Polytechnic Institute of the Rio de Janeiro State University (IPRJ/UERJ). The equipment consists of: (i) a single-phase asynchronous induction motor (1750 rpm, 1/4 HP) coupled to a gear reducer (ratio 37:1); (ii) a lower plate of machinable stainless steel (radius *R*_1_ = 150.00 mm) driven by the motor; (iii) an upper plate of the same dimensions, fixed, supported on a central shaft with a tapered bearing; (iv) a dynamometer connected to the upper plate by means of a thin line, for measuring the viscous force; and (v) a thermocouple for monitoring the fluid temperature (Figure 2).

**Figure 2:**
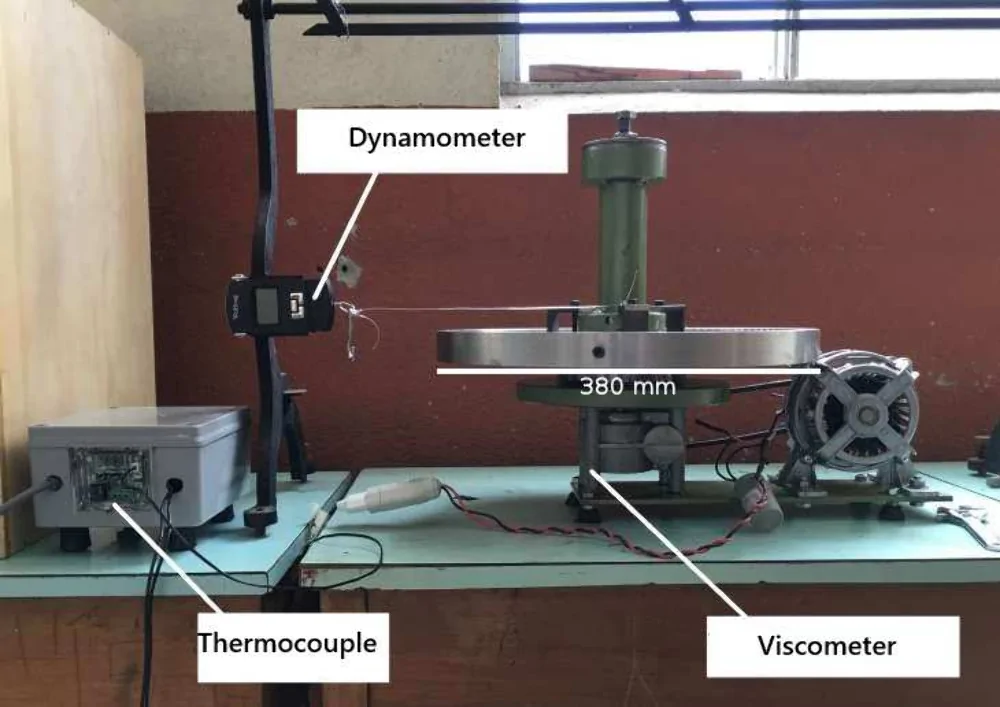
General view of the constructed parallel plate viscometer illustrating the motor and reducer, driven lower plate, fixed upper plate with dynamometer and thermocouple.

The spacing between the plates (*h*) is adjustable by means of a micrometric screw, ranging from 1.25 mm to 6.25 mm in steps of 1.25 mm.

The plate radius (*R*_1_ = 150.00 mm) was intentionally selected to increase the sensitivity of the proposed low-cost viscometer. According to Macosko,^26^ the torque developed in a parallel-plate geometry is proportional to the fourth power of the plate radius (*M* ∝ *R* ^4^). Therefore, the larger plate radius increases the measurable torque, enabling reliable force measurements with a manual dynamometer. Furthermore, for the maximum plate spacing (*h* = 6.25 mm), the adopted geometry provides a ratio of *R*/*h* = 24, satisfying the condition that the plate radius should be much larger than the gap to minimize edge effects. As discussed by Schlichting and Gersten,^27^ the larger radius also allows the desired shear rates to be achieved at relatively low angular velocities, contributing to the maintenance of laminar flow conditions.

### Mathematical Modeling

For a Newtonian fluid in laminar flow, with no-slip condition at the plates and neglecting edge effects, the shear stress *τ* at a distance *r* from the center is given by Newton’s law of viscosity.^1,2^ The experience of our group in high-precision computational modeling^9^ and numerical simulation^10^ was fundamental to ensure the mathematical rigor of this analysis.

1 $$\tau =\mu \frac{du}{dy}\approx \mu \frac{\Omega r}{h}$$

Integrating the differential torque *dM* = *r*·*τ*·*dA* over the annular area between the internal shaft radius *R*_0_ = 20.00 mm and the external plate radius *R*_1_ = 150.00 mm, the total torque *M* on the upper plate is obtained:

2 $$M=\int_{R_{0}}^{R_{1}}r\cdot \left(\mu \frac{\Omega r}{h}\right)\cdot \left(2\pi rdr\right)=\frac{2\pi \mu \Omega }{h}\int_{R_{0}}^{R_{1}}r^{3}\mathrm{dr}=\frac{\pi \mu \Omega }{2h}\left(R_{1}^{4}-R_{0}^{4}\right)$$

Isolating the dynamic viscosity *μ*, we have

3 $$\mu =\frac{2hM}{\pi \Omega \left(R_{1}^{4}-R_{0}^{4}\right)}$$

The torque *M* is obtained experimentally from the force *F* measured by the dynamometer, applied at a distance *r*_*M*_ = 52.25 mm from the shaft center: M = *F* · *r*_*M*_. The effective force is calculated as *F* = *F*_*L*_ – *F*_*I*_, where *F*_*L*_ is the force with the lubricant and *F*_*I*_ is the friction force of the equipment without fluid (“empty” measurements).

### Experimental Procedure

Two commercial lubricants were studied: a semi-synthetic SAE 15W40 and a mineral SAE 20W50. The procedure consisted of the following:

1. Adjust the spacing *h* between the plates (6.25, 5.00, 3.75, 2.50, 1.25 mm).
2. Measure the force *F*_*I*_ (without fluid) for 30 seconds, recording the minimum and maximum values.
3. Add the lubricant between the plates and measure the force *F*_*L*_, recording temperature and force values. The required sample volume was calculated as *V* = *π*(*R*_1_^2^ – *R*_0_^2^)*h*, resulting in approximately 433.9 mL for the maximum gap (*h* = 6.25 mm). Temperature was continuously monitored to minimize thermal effects.
4. Calculate *F* = *F*_*L*_ – *F*_*I*_ and the torque M = *F* · *r*_*M*_.
5. Calculate *μ* using eq 3, with uncertainty propagation according to GUM methodology.^11^

All measurements were performed at controlled room temperature (17.4 °C to 19.7 °C), with continuous monitoring by thermocouple.

### Shear Rate Calculation

The maximum shear rate in the parallel plate flow occurs at the outer edge of the plate (*r* = *R*_1_) and is given by^1^

4 $${\mathrm{\gamma ̇}}_{\max}=\frac{\Omega R_{1}}{h}$$

With *Ω* = 4.95 s^–1^ and *R*_1_ = 0.150 m, the rates for each spacing *h* are calculated. Table 1 presents the results.

**Table 1:** Maximum Shear Rates for Each Plate Spacing

**Spacing** *h* **(mm)**	**Shear rate** *γ* **˙**_**max**_ **(s** ^**1**^ **)**
6.25	119
5.00	149
3.75	198
2.50	297
1.25	594

It is observed that the shear rate varies from 119 s^1^ (*h* = 6.25 mm) to 594 s^1^ (*h* = 1.25 mm). As will be discussed below, this variation plays a fundamental role in the observed rheological behavior of the lubricants.

### Modified Rotational Reynolds Number Calculation

To ensure the validity of the rheological measurements, it is essential to evaluate the relative importance of inertial effects compared with viscous forces, which is quantified by the modified rotational Reynolds number (*Re*_*ω*_). In parallel-plate rheometers, fluid inertia may generate secondary flows induced by centrifugal effects, causing the fluid to move radially outward along the rotating plate and inward along the stationary plate. These instabilities increase energy dissipation, resulting in a measured torque greater than the ideal shear torque and, consequently, an error in the calculated viscosity.^28^

For the parallel-plate geometry, the modified rotational Reynolds number is given by

5 $$Re_{\omega }=\frac{\rho \Omega h^{2}}{\mu }$$

where *ρ* is the fluid density, *Ω* is the angular velocity, *h* is the plate gap, and *μ* is the dynamic viscosity.

According to Spagnolie,^28^ in order to keep the error in the measured torque below approximately 1%, the modified rotational Reynolds number should remain below the critical value of *Re*_crit_ = 4. Above this threshold, the flow is no longer purely laminar, and inertial effects begin to significantly influence the rheometer response.

Using the experimental angular velocity of *Ω* = 4.95 rad s^–1^ and the specific densities of the lubricants at 20 °C (*ρ*_15W40_ = 890 kg m^–3^ and *ρ*_20W50_ = 880.3 kg m^–3^), the modified rotational Reynolds number (*Re*_*ω*_) was calculated for each lubricant at all tested plate gaps using the experimentally determined dynamic viscosities (*μ*). The resulting values are presented in Table 2.

**Table 2:** Calculated Values of the Modified Rotational Reynolds Number (*Re*_*ω*_) (*Re*_*ω*_ Is Dimensionless)

**Plate gap** *h* (**mm**)	*Re*_*ω*_ (**SAE 15W40**)	*Re*_*ω*_ (**SAE 20W50**)
6.25	0.506	0.436
5.00	0.324	0.287
3.75	0.177	0.175
2.50	0.071	0.085
1.25	0.009	0.027

As shown in Table 2, all calculated values of *Re*_*ω*_ are well below the critical limit of 4. Even for the largest plate gap (*h* = 6.25 mm), where inertial effects are expected to be most pronounced, the highest value obtained was approximately *Re*_*ω*_ = 0.51. These results demonstrate that the experiments were conducted entirely within the laminar and hydrodynamically stable flow regime, ensuring that the measured torque and, consequently, the viscosity curves were not affected by inertial instabilities or the development of secondary flows.

### Brinkman Number and Viscous Dissipation Analysis

To assess the potential influence of viscous heating on the measured viscosity, the Brinkman number (*Br*) was estimated for the most severe operating condition, namely the smallest plate gap (*h* = 1.25 mm) and the highest shear rate (*γ̇*_max_ = 594 s^–1^). The Brinkman number represents the ratio between viscous heat generation and conductive heat transfer, and is defined as

6 $$\mathrm{Br}=\frac{\mu v^{2}}{\kappa \Delta T}$$

where *μ* is the dynamic viscosity, *v* = *ΩR*_1_ is the tangential velocity at the outer edge of the rotating plate, *κ* is the thermal conductivity of the lubricant, and *ΔT* is a characteristic temperature rise. eq 6 was used to estimate the relative importance of viscous dissipation.

For the SAE 20W50 lubricant at *h* = 1.25 mm, the experimentally determined dynamic viscosity was *μ* = 0.25 Pa·s, the tangential velocity at the plate edge is *v* = *ΩR*_1_ = 4.95 × 0.150 = 0.7425 m s^–1^, and the thermal conductivity of typical mineral-based lubricants is approximately *κ* ≈ 0.14 W m^–1^ K^–1^.^31^ Considering a characteristic temperature rise of *ΔT* = 1 K (a conservative estimate for the small temperature variations observed during the experiments, which ranged from 17.4 °C to 19.7 °C), the Brinkman number is

7 $$\mathrm{Br}=\frac{0.25\times {\left(0.7425\right)}^{2}}{0.14\times 1}\approx \frac{0.25\times 0.551}{0.14}\approx \frac{0.138}{0.14}\approx 0.99$$

For the SAE 15W40 lubricant at the same condition (*μ* = 0.78 Pa·s), the Brinkman number is

8 $$\mathrm{Br}=\frac{0.78\times {\left(0.7425\right)}^{2}}{0.14\times 1}\approx \frac{0.78\times 0.551}{0.14}\approx \frac{0.430}{0.14}\approx 3.07$$

The Brinkman number provides an indication of the relative importance of viscous dissipation. For *Br* ≪ 1, conduction dominates and viscous heating is negligible. For *Br* ∼ 1 or greater, viscous dissipation may become significant and should be considered.

The calculated values of *Br* ≈ 0.99 for SAE 20W50 and *Br* ≈ 3.07 for SAE 15W40 suggest that viscous heating is not entirely negligible, particularly for the SAE 15W40 lubricant at the smallest gap. However, the observed temperature variation during the experiments was only ∼2 K (from 17.4 °C to 19.7 °C), and the temperature dependence of viscosity for engine oils is typically 2 – 3% per Kelvin. Thus, a temperature rise of approximately 1 K would produce a viscosity variation of only 2 – 3%, which is comparable to or smaller than the experimental uncertainties reported in this work.

Moreover, the distinct behavior observed for the two lubricants — increasing apparent viscosity for SAE 15W40 and decreasing apparent viscosity for SAE 20W50 with reduced plate spacing — cannot be explained by viscous heating alone. Viscous heating would cause a reduction in viscosity for both lubricants, whereas the SAE 15W40 exhibited an increase. This indicates that the dominant effects at small gaps are related to non-Newtonian rheology and/or non-parallelism of the plates, rather than thermal dissipation.

Nevertheless, for future work, it is recommended to implement a controlled temperature system to further minimize thermal effects and to enable the study of viscosity as a function of temperature.

### Uncertainty Propagation

The combined standard uncertainty of viscosity was calculated from eq 3, considering the sources: spacing *h*, torque *M*, rotation *Ω*, radii *R*_0_ and *R*_1_. The law of uncertainty propagation was used:^11^

9 $$\sigma_{\mu }^{2}={\left(\frac{\partial \mu }{\partial h}\right)}^{2}\sigma_{h}^{2}+{\left(\frac{\partial \mu }{\partial M}\right)}^{2}\sigma_{M}^{2}+{\left(\frac{\partial \mu }{\partial \Omega }\right)}^{2}\sigma_{\Omega }^{2}+{\left(\frac{\partial \mu }{\partial R_{0}}\right)}^{2}\sigma_{R_{0}}^{2}+{\left(\frac{\partial \mu }{\partial R_{1}}\right)}^{2}\sigma_{R_{1}}^{2}$$

where *σ*_*h*_, *σ*_*M*_, *σ*_*Ω*_, *σ*_*R*_0__, and *σ*_*R*_1__ are the standard uncertainties of the plate spacing, torque, angular velocity, inner radius, and outer radius, respectively. The uncertainty of the torque is calculated from the uncertainties of the measured force (*F*) and lever arm (*r*_*M*_) as $\sigma_{M}=\sqrt{{\left(r_{M}\sigma_{F}\right)}^{2}+{\left(F\sigma_{r_{M}}\right)}^{2}}$ . All units are consistent with the SI units reported in Table 3.

**Table 3:** Uncertainties of Input Quantities

**Quantity**	**Symbol**	**Nominal value**	**Standard** **uncertainty** *σ*
Angular velocity	*Ω*	4.95 s^1^	*σ*_*Ω*_ = ±0.09 s^1^
Inner radius	*R*_0_	20.00 mm	*σ*_*R*_0__ = ±0.05 mm
Outer radius	*R*_1_	150.00 mm	*σ*_*R*_1__ = 0.05 mm
Lever arm	*r*_*M*_	52.25 mm	*σ*_*r*_*M*__ = ±±0.05 mm
Measured force	*F*	(variable)	*σ*_*F*_ = ±0.1 to 2 N
Plate spacing	*h*	(variable)	*σ*_*h*_ = ±0.05 mm
Torque	*M*	(variable)	$\sigma_{M}=\sqrt{{\left(r_{M}\sigma_{F}\right)}^{2}+{\left(F\sigma_{r_{M}}\right)}^{2}}$ (calculated, N·m)

The partial derivatives are obtained directly from Equation 3. The uncertainties of the input quantities are summarized in Table 3.

## Results

This section presents the experimental results obtained for the two lubricants studied.

### Lubricant SAE 15W40

Table 4 presents the experimental dynamic viscosity values obtained for the SAE 15W40 lubricant for the five plate spacings (*h*) and their respective measurement temperatures.

**Table 4:** Experimental Dynamic Viscosity of SAE 15W40 Lubricant

**Spacing** *h* **(mm)**	**Temperature (**°**C)**	**Viscosity** *μ* ± *σ*_*μ*_ (Pa·s)
6.25	18.10	0.34 ± 0.02
5.00	18.30	0.34 ± 0.02
3.75	18.80	0.35 ± 0.01
2.50	18.90	0.39 ± 0.01
1.25	19.10	0.78 ± 0.03

It is observed that for larger spacings (*h* ≥ 5.00 mm), the viscosity remains stable around 0.34 Pa·s. For *h* = 3.75 mm, the value is still close (0.35 Pa·s). However, as the spacing decreases to *h* = 2.50 mm and especially to *h* = 1.25 mm, the calculated viscosity increases significantly, reaching 0.78 Pa·s (Figure 3).

**Figure 3:**
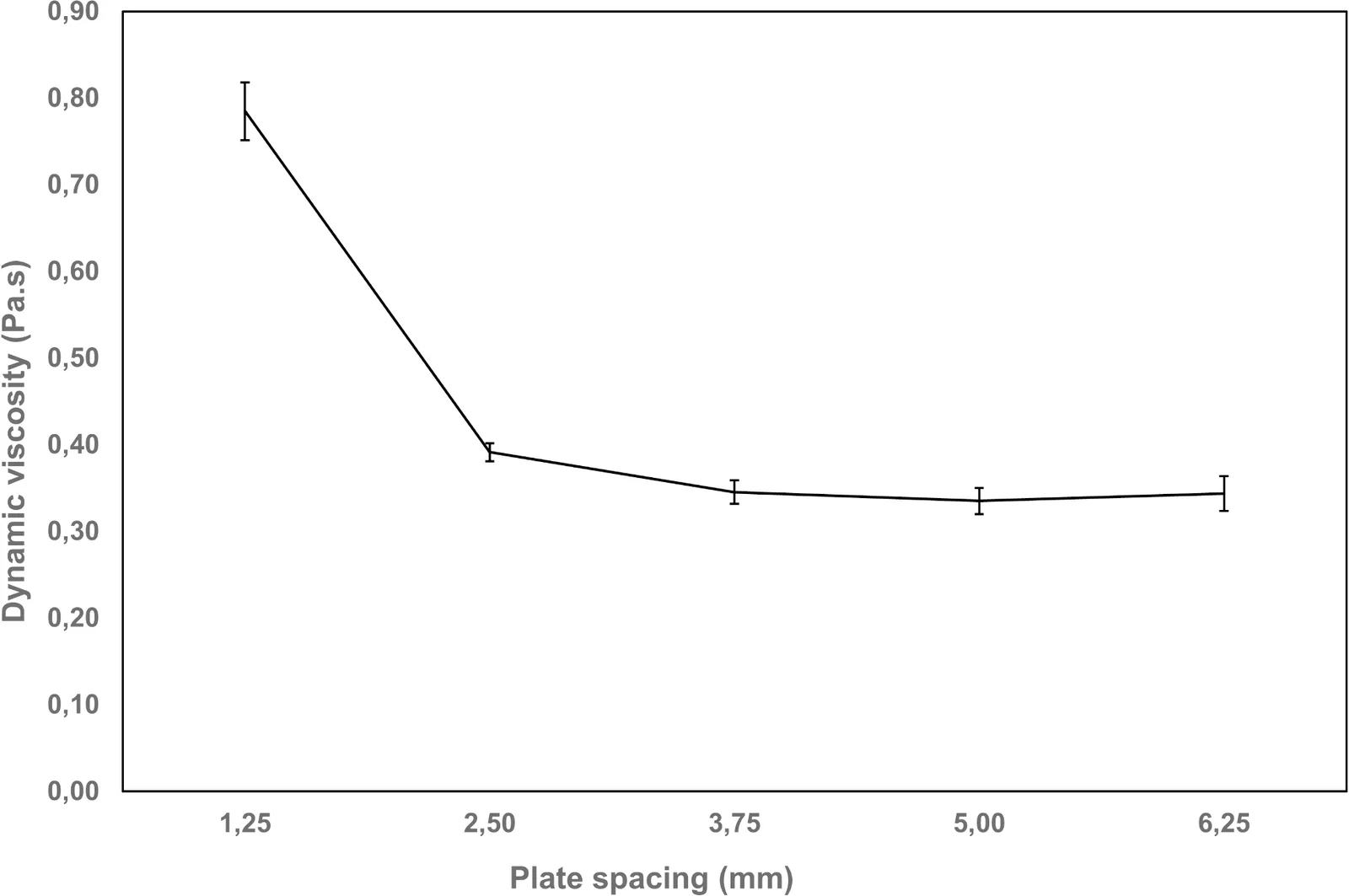
Viscosity of SAE 15W40 lubricant experimentally determined as a function of plate spacing.

### Lubricant SAE 20W50

Table 5 presents the experimental results for the SAE 20W50 lubricant.

**Table 5:** Experimental Dynamic Viscosity of SAE 20W50 Lubricant

**Spacing** *h* **(mm)**	**Temperature (**°**C)**	**Viscosity** *μ* ± *σ*_*μ*_ (Pa·s)
6.25	18.20	0.39 ± 0.01
5.00	18.50	0.38 ± 0.01
3.75	18.70	0.35 ± 0.01
2.50	19.00	0.32 ± 0.01
1.25	19.20	0.25 ± 0.00

Unlike observed for SAE 15W40, the SAE 20W50 lubricant shows a decreasing viscosity trend with reduced plate spacing (Figure 4).

**Figure 4:**
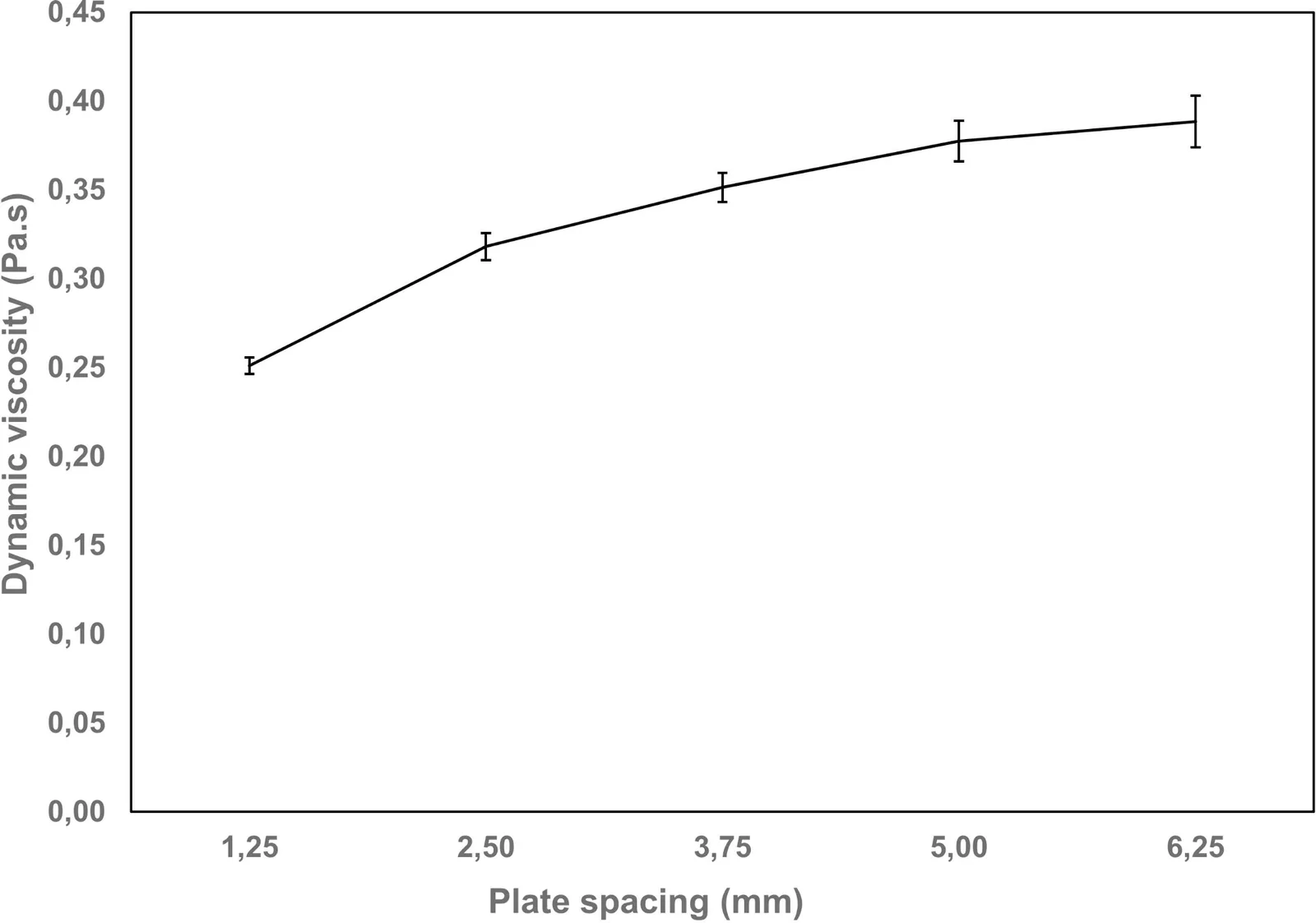
Viscosity of SAE 20W50 lubricant experimentally determined as a function of plate spacing.

### Comparison with Literature Data

Table 6 presents the comparison of experimental results for SAE 15W40 with data from Anton Paar.^3^

**Table 6:** Comparison between Experimental Viscosities and Anton Paar Data for SAE 15W40

**Temperature (°**C**)**	**Spacing** *h* **(**mm**)**	**Anton Paar Viscosity**(Pa·s)	**Experimental Viscosity**(Pa·s)
18.10	6.25	0.33	0.34 ± 0.02
18.30	5.00	0.32	0.34 ± 0.02
19.10	1.25	0.30	0.78 ± 0.03

Figure 5 shows that the experimental results for *h* = 6.25 mm and *h* = 5.00 mm are consistent with the reference data.^3^ For *h* = 1.25 mm, the experimental value deviates significantly.

**Figure 5:**
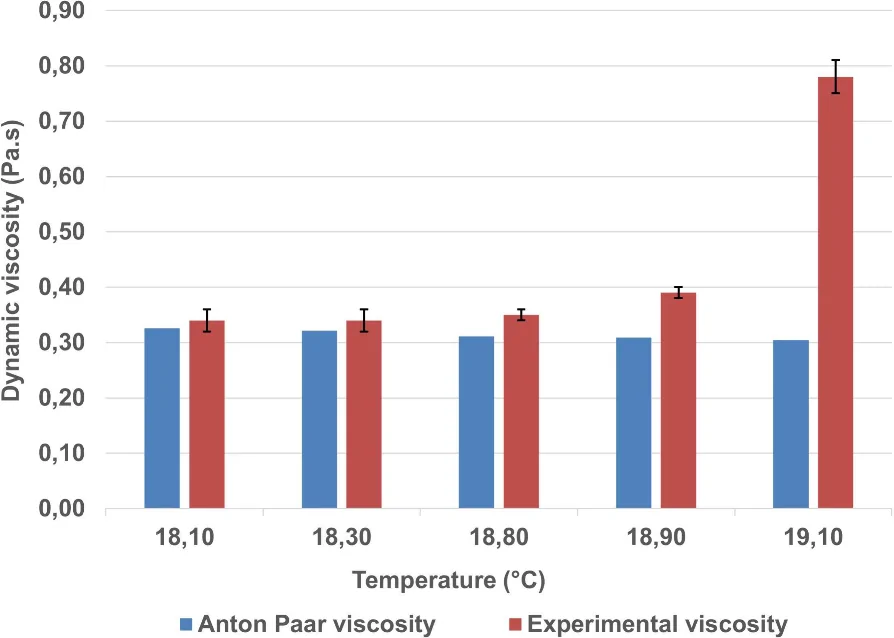
Comparison between the viscosity of SAE 15W40 lubricant experimentally determined and literature data from Anton Paar.^3^

### Comparison between the Two Lubricants

Figure 6 presents the direct comparison between the two lubricants studied.

**Figure 6:**
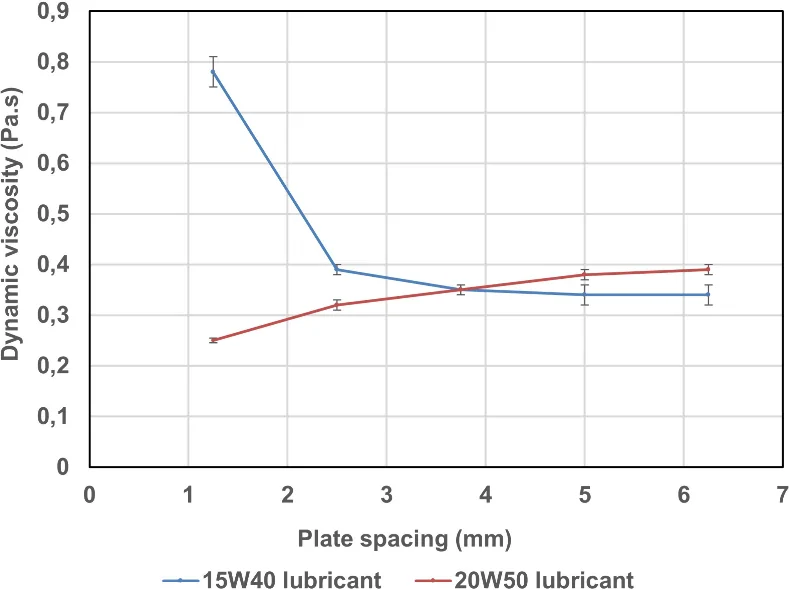
Viscosity of SAE 15W40 vs SAE 20W50 as a function of plate spacing.

For *h* = 6.25 mm, the viscosity of SAE 20W50 (0.39 Pa·s) is higher than that of SAE 15W40 (0.34 Pa·s), as expected by the SAE classification. As the spacing decreases, the curves converge.

## Discussion

This section elaborates on the results presented above and discusses their significance and relationship to the existing literature.

The experimental results clearly demonstrate that the plate spacing (*h*) exerts a significant influence on the calculated viscosity, especially for reduced *h* values. As shown in Table 1, the reduction of *h* from 6.25 mm to 1.25 mm raises the maximum shear rate from 119 s^1^ to 594 s^1^. This variation is sufficient to induce non-Newtonian behavior in lubricants containing viscosity modifier additives.^12,13^

For the spacing of 1.25 mm, the maximum shear rate reaches approximately 594 s^1^. This is consistent with findings by Bhattad et al.,^24,25^ who reviewed the influence of shear rate on the viscosity of complex fluids, noting that many industrial fluids exhibit shear-thinning or shear-thickening behavior at rates exceeding 500 s^1^. Studies indicate that multigrade oils with polymeric additives may exhibit pseudoplastic behavior at shear rates in the range of 500 to 1000 s^1^,^14,15^ which partially explains the deviations observed for the SAE 15W40 lubricant. The increase in calculated viscosity for this oil at low *h* suggests that the Newtonian model, which assumes constant viscosity, is no longer valid under these conditions.

The distinct behavior of the SAE 20W50 lubricant, which showed a decrease in viscosity with the reduction of *h*, may be associated with differences in its formulation. SAE 15W40 is a semi-synthetic oil, while SAE 20W50 is mineral. The distinct rheological response suggests that the former has a higher concentration or different types of viscosity modifier polymers, which can elongate in the flow direction under high shear rates, reducing flow resistance.^12^

The effect observed for SAE 15W40 was opposite (increase in viscosity), which is consistent with a shear-thickening behavior induced by the high shear rates at small gaps. A quantitative estimate of plate non-parallelism (bearing clearance of 0.04 mm, resulting in a maximum edge misalignment of 0.035 mm) indicates that geometric imperfections contribute only ∼2.8% to the viscosity uncertainty at *h* = 1.25 mm, which is smaller than the observed increase. However, other geometric factors such as plate non-flatness or additional machining imperfections may also contribute to the deviations at small gaps. Therefore, while non-Newtonian rheological effects appear to play a significant role, the contribution of geometric artifacts cannot be entirely ruled out.

However, the substantial viscosity deviations observed at smaller gap dimensions (*h* ≤ 3.75 mm), where the measured values reach up to 0.78 ± 0.03 Pa·s for the SAE 15W40 oil, may also be interpreted in light of effects associated with spatial confinement and boundary layers. Recent research on complex media subjected to intense shear and high spatial confinement suggests that restricted gap domains can induce distinct flow regimes and significantly modify effective transport properties.^29,30^ In this context, as the gap width approaches the characteristic scales associated with boundary layers, the measured rheological response may reflect not only the non-Newtonian effects of the fluid but also changes in the flow field resulting from spatial confinement. This interpretation provides a possible additional physical explanation for the deviations observed at small gap dimensions, without excluding the residual contribution of geometric imperfections.

Although Zamankhan’s methodology employs granular metafluids rather than molecular lubricants, the underlying concept of confinement-induced transport transitions provides a valuable analogy for interpreting the present viscometric data.

Furthermore, restricted gap domains may alter local energy dissipation mechanisms through shear-induced transitions. Studies of granular metafluids subjected to extensional and shear deformations have reported strongly anisotropic stress patterns and deviations from Newtonian behavior under confined and highly sheared conditions.^30^ Although these systems differ from the lubricants investigated here, such findings provide a useful physical framework for interpreting the present results, suggesting that strong confinement and high shear rates may contribute to changes in the apparent viscosity through modifications of the local flow and energy-dissipation mechanisms. Thus, the deviations observed at small *h* may indicate not only a mechanical limitation of the apparatus, but also the onset of a regime in which the assumptions of Newtonian transport become progressively less applicable.

Beyond the intrinsic flow modifications arising from confinement, additional experimental sources of uncertainty were also examined, including the influence of the free surface and edge effects.

The free-surface stability was also considered as a potential source of uncertainty. The raised peripheral edge of the lower plate creates a drowned-edge configuration that stabilizes the meniscus and prevents fluid overflow during operation, which is particularly important due to the large plate diameter adopted. However, according to Macosko,^26^ this configuration may introduce an additional torque contribution when the fluid extends beyond the upper plate edge. This contribution can be estimated using the correction factor *M*/*M*_0_ = 1 + 1.9*h*/*R*, where *M*_0_ is the ideal torque in the absence of edge effects, representing a systematic effect that limits the current design and should be considered in future improvements of the instrument.

Another relevant factor is the possible viscous heating of the fluid. At high shear rates (close to 600 s^1^), viscous dissipation can locally raise the temperature of the lubricant, reducing its actual viscosity.^16^ This effect could explain the decreasing trend observed for SAE 20W50. However, as demonstrated by the Brinkman number analysis, the increase seen for SAE 15W40 at small gaps cannot be attributed to viscous heating alone, since thermal dissipation would reduce viscosity in both cases. This reinforces the interpretation that non-Newtonian rheological effects, particularly shear-thickening of the semi-synthetic oil, are the dominant mechanism at small gaps, though geometric artifacts may also contribute.

The Brinkman number analysis presented above indicates that viscous heating is not entirely negligible, with *Br* ≈ 0.99 for SAE 20W50 and *Br* ≈ 3.07 for SAE 15W40 at *h* = 1.25 mm. However, the distinct rheological responses of the two lubricants — shear-thickening for SAE 15W40 versus shear-thinning for SAE 20W50 — cannot be explained by viscous heating alone, since thermal dissipation would cause a viscosity decrease in both cases. This supports the interpretation that the dominant effects at small gaps are related to non-Newtonian rheology and/or non-parallelism of the plates.

The rheological behavior observed for both oils highlights the importance of considering the interplay between shear rate, fluid composition, and temperature when designing and validating viscometric measurements. The SAE 15W40, being a semi-synthetic oil with a more complex additive package, appears more susceptible to shear-thickening under the high shear rates induced by small plate spacings. In contrast, the simpler formulation of the mineral SAE 20W50 results in a more pronounced shear-thinning effect.

The methodology developed in this work for viscometer validation is aligned with the consolidated experience of our research group in experimental and modeling methods for tribological analysis, as documented in recent publications on friction in cam-tappet systems.^4,5,17^ Furthermore, the group’s expertise in the application of mathematical and empirical modeling for solving complex engineering problems,^6,9^ as well as proficiency in numerical simulation and experimental validation,^10,18^ were fundamental to ensure the rigor and reliability of the results presented here.

The ability to build and validate low-cost instrumentation, as already demonstrated in publications in the journal *Measurement*,^19^ was essential for the success of this work. In addition, the group’s experience in advanced non-destructive characterization techniques by X-ray fluorescence^20,21^ and computational modeling^22,23^ corroborates the multidisciplinary approach adopted.

The validation of the equipment, demonstrated by the agreement between the experimental results for *h* = 6.25 mm and *h* = 5.00 mm with the Anton Paar data^3^ (Table 6 and Figure 5), indicates that the constructed viscometer is capable of providing reliable measurements when operated in the range of lower shear rates (*h* ≥ 5 mm). Under these conditions, the maximum shear rate is 149 s^1^ or lower, a range in which the studied lubricants behave as Newtonian.

## Conclusions

This work presented the design, construction and validation of a low-cost parallel plate rotational viscometer for characterization of automotive lubricants. Based on the results obtained, the following conclusions can be established:

1. The constructed equipment was successfully validated for determining the dynamic viscosity of SAE 15W40 and 20W50 lubricants, provided that it is operated with plate spacing *h* ≥ 5 mm, a condition in which the experimental results (0.34 ± 0.02 Pa·s for 15W40) presented agreement with the Anton Paar literature data^3^ within the uncertainty bars.
2. The plate spacing exerts a strong influence on the results: for *h* = 6.25 mm and 5.00 mm the values are stable and precise; for *h* = 3.75 mm deviations begin to appear; and for *h* = 2.50 mm and especially *h* = 1.25 mm, the deviations become unacceptable, with the calculated viscosity for SAE 15W40 reaching 0.78 ± 0.03 Pa·s, more than double the reference value.
3. The analysis of shear rates (ranging from 119 s^1^ to 594 s^1^) showed that the deviations for small *h* are associated with the transition to non-Newtonian behavior, confirming that the Newtonian fluid hypothesis is valid only for the largest plate spacings tested.
4. The two lubricants presented distinct rheological behaviors: SAE 15W40 showed an increase in calculated viscosity with the reduction of *h*, while SAE 20W50 showed a decrease. This difference probably reflects variations in their formulations (semi-synthetic vs. mineral) and in the response of viscosity modifier additives to different shear rates, although geometric artifacts such as non-parallelism and non-flatness of the plates may also contribute to the deviations at small gaps.

The key quantitative findings are summarized in Table 7.

**Table 7:** Summary of Key Quantitative Results

**Lubricant**	**Spacing** *h* **(mm)**	**Viscosity** *μ* (Pa·s)	**Deviation from Anton Paar (%)**
SAE 15W40	6.25	0.34 ± 0.02	+3.0%
SAE 15W40	1.25	0.78 ± 0.03	+160.0%
SAE 20W50	6.25	0.39 ± 0.01	N/A
SAE 20W50	1.25	0.25 ± 0.00	N/A

Note: Reference data for SAE 20W50 was not available in the Anton Paar database used for comparison. However, the measured values for SAE 20W50 are consistent with the expected behavior for this oil grade based on its SAE classification (higher viscosity than SAE 15W40 at 6.25 mm).

These results demonstrate that while the low-cost viscometer is validated for reliable viscosity measurements (within ∼3% of a reference standard) for *h* ≥ 5 mm, its use outside this range leads to significant and non-systematic errors, which can exceed 160% for SAE 15W40. This underscores the critical importance of establishing and adhering to a reliable operational range for such instruments.

The Brinkman number analysis indicated that viscous heating is not entirely negligible at the smallest plate gap (*h* = 1.25 mm), with *Br* ≈ 0.99 for SAE 20W50 and *Br* ≈ 3.07 for SAE 15W40. However, the distinct rheological responses observed — shear-thickening for SAE 15W40 versus shear-thinning for SAE 20W50 — cannot be attributed to viscous heating alone, which would reduce the viscosity in both cases. Therefore, the dominant effects at small gaps are attributed to non-Newtonian rheology and/or non-parallelism of the plates.

As future work, it is suggested: (i) the instrumentation of the equipment with load cells and temperature sensors coupled to microcontrollers (Arduino) for automation of measurements; (ii) the implementation of a controlled heating system to study the variation of viscosity with temperature; (iii) the machining of the upper plate to allow larger spacings; and (iv) the study of the behavior of contaminated or aged lubricants; and (v) the implementation of multi-scale computational fluid dynamics (CFD) simulations of the viscometer’s internal dynamics.

## Abbreviations

SAE: Society of Automotive Engineers
GUM: Guide to the Expression of Uncertainty in Measurement
RPM: rotations per minute
CV: horsepower
IPRJ: Polytechnic Institute
UERJ: Rio de Janeiro State University
